# A four-miRNA serum panel as a diagnostic biomarker for prostate cancer: an exploratory and validation study

**DOI:** 10.3389/fonc.2026.1866712

**Published:** 2026-07-27

**Authors:** Zuodong Yang, Zixiang Lai, Jiaodie Xie, Siwei Chen, Zhenjian Ge, Yongqing Lai, Hang Li

**Affiliations:** 1 Department of Urology, Peking University Shenzhen Hospital, Shenzhen, China; 2 Institute of Urology, Shenzhen Peking University-The Hong Kong University of Science and Technology Medical Center, Shenzhen, China; 3 Shenzhen Clinical Research Center for Urology and Nephrology, Shenzhen, China; 4 Shenzhen University Health Science Center, Shenzhen, China; 5 Shenzhen University Medical School, Shenzhen, China; 6 College of basic Medicine, Xinjiang medical university, Wulumuqi, China; 7 Peking University Shenzhen Hospital, Shenzhen, China; 8 Department of Urology, Shenzhen Xinhua Hospital, Shenzhen, China

**Keywords:** bioinformatics, biomarker, diagnosis, miRNA, prostate cancer, PSA, serum biomarker

## Abstract

**Background:**

Prostate-specific antigen (PSA) is widely used for prostate cancer (PC) screening, but its relatively low diagnostic accuracy may compromise early diagnosis and clinical management. There is an urgent need to identify convenient, cost-effective, and non-invasive diagnostic methods to reduce the rates of false positives and false negatives associated with PSA testing for prostate cancer. Serum miRNAs hold great promise as diagnostic tumor biomarkers. This study aimed to identify a novel serum miRNA panel for PC detection, with the goal of addressing the two major limitations of PSA: high false-positive and false-negative rates.

**Materials and methods:**

This study was conducted in three phases: biomarker screening, training, and validation. First, candidate miRNAs associated with prostate cancer were screened using the PubMed and ENCORI databases. Subsequently, real-time quantitative reverse transcription polymerase chain reaction (RT-qPCR) was performed to analyze miRNA expression in serum samples from the training group: 28 PC and 28 normal controls (NC), and the validation group: 84 PC and 84 NC, and to identify differentially expressed miRNAs. We assessed their diagnostic performance using receiver operating characteristic (ROC) curves and the area under the curve (AUC). We then constructed miRNA panel diagnostic models comprising different numbers of miRNAs to balance diagnostic performance and detection costs. Finally, bioinformatics analyses were performed to identify their target genes and functional pathways.

**Results:**

During the screening phase, 10 candidate miRNAs associated with PC were identified. In the training phase, 6 candidate miRNAs showed significant differential expression in PC compared with NC. In the validation phase, 5 candidate miRNAs (miR-101-3p, miR-154-5p, miR-199a-5p, miR-145-5p, and miR-30-5p) demonstrated favorable diagnostic performance for PC. After balancing diagnostic performance and detection costs, we ultimately selected the optimal 4-miRNA panel (miR-101-3p, miR-154-5p, miR-199a-5p, and miR-145-5p) as the final diagnostic biomarker panel for PC. This panel showed favorable diagnostic performance (AUC = 0.825, sensitivity = 83.33%, specificity = 69.05%). Bioinformatics analysis indicated that the *NFASC* gene may serve as a common target of this 4-miRNA panel and was significantly enriched in cancer-related pathways, suggesting its potential involvement in the development and progression of PC.

**Conclusion:**

A panel consisting of miR-101-3p, miR-154-5p, miR-199a-5p, and miR-145-5p exhibited excellent diagnostic efficacy for PC. This serum miRNA panel possesses translational potential and provides a novel strategy to address the two major limitations of PSA testing: high false-positive and false-negative rates.

**Clinical trial registration number:**

https://www.chictr.org.cn/showproj.html?proj=187882, identifier ChiCTR2200066840.

## Introduction

PC is a prevalent malignant tumor in males. Globally, it ranks second among all male malignancies, preceded only by lung cancer, and accounts for 7.3% of all cancer cases (1). Globally, there are approximately 1.4 million new cases and 390, 000 related deaths each year (2). Both the United States and China present high incidence rates and a sustained upward trend (3, 4), making PC a major health threat to middle-aged and elderly males (5). PC is notably insidious: it is often asymptomatic in the early stages, with only a small proportion of patients presenting with urinary abnormalities, and symptoms typically manifest in the advanced stages (6). Once metastasis occurs, the disease is largely incurable, with a 5-year overall survival (OS) rate of only 30%–40% (7). The increasing incidence of PC is associated with population aging, lifestyle modifications, and widespread screening (3), while its core pathogenesis remains elusive (5). PC poses a severe and persistent threat to male health worldwide.

PSA is a glycoprotein widely applied in PC screening (8–10). On the one hand, PSA yields a high rate of false-positive results. Its positive predictive value is approximately 19–35% (11–13). This indicates that many patients present false-positive outcomes, which often necessitate further diagnostic examinations including needle biopsy and MRI (9, 14), thereby exposing individuals to unnecessary psychological burden, excessive testing, and even physical injury (15, 16). Consequently, it remains controversial whether the benefits of PSA screening outweigh its potential harms (17), and there is an urgent need to develop a convenient, cost-effective, and non-invasive diagnostic approach to mitigate the false-positive rate of PSA. On the other hand, PSA yields a high incidence of false-negative results (11–13). Its false-negative rate ranges from 11% to 39% (18–21). When the PSA cutoff value is set at 3, 4, or 5 ng/ml, the corresponding false-negative rates are 41%, 56%, and 67%, respectively (22). When the PSA test yields negative findings, patients usually receive active surveillance and regular follow-up. A false-negative PSA result may lead to missed diagnosis and delayed treatment of PC, thereby contributing to tumor progression and even advanced disease. Consequently, affected individuals are at risk of adverse outcomes including unfavorable prognosis, elevated treatment costs, diminished quality of life, and reduced OS. Therefore, it is urgent to establish a convenient, cost-effective and non-invasive diagnostic strategy to lower the false-negative rate of PSA.

Circulating microRNAs (miRNAs), as a group of stable non−coding small RNA molecules, participate in almost all cellular regulatory processes. Dysregulated expression of these molecules, including upregulation and downregulation, is closely linked to the progression of numerous human diseases (23, 24). Dysfunction of mature miRNAs can disrupt the expression of oncogenes and tumor suppressor genes (25). miRNAs can be detected non-invasively in peripheral blood and present superior characteristics, including tissue− and tumor−specific expression patterns and favorable detection reproducibility (24, 26), which endows them with promising application potential in clinical detection. Recent studies have verified that serum miRNAs act as promising biomarkers for multiple malignancies, including renal cell carcinoma (27) and bladder cancer (28). In summary, circulating miRNAs hold great promise as novel biomarkers for PC detection.

We hypothesize that a panel of serum miRNAs presents high sensitivity and specificity in PC and can act as a reliable tumor biomarker for this disease. Therefore, the aim of this study is to identify a novel miRNA panel for PC detection.

## Materials and methods

### Statement on research participants and ethics

With the approval of the Ethics Committee of Peking University Shenzhen Hospital, we enrolled 224 participants, including 112 cases of PC and 112 NC. All participants and/or their legal guardians signed informed consent forms, and the serum sampling process strictly adhered to the relevant guidelines issued by the Ethics Committee. Inclusion criteria: (1) All prostate cancer patients were pathologically confirmed via postoperative biopsy; (2) Complete clinical data and laboratory test results were available; (3) Participants were able to cooperate with sample collection and signed informed consent voluntarily; (4) Age range was limited to adult males. Exclusion criteria: (1) Patients who had received surgery, radiotherapy, chemotherapy, endocrine therapy or other anti-tumor treatments before blood sampling; (2) Individuals with other malignant tumors, severe organ dysfunction or systemic inflammatory diseases; (3) Patients with benign prostate diseases such as benign prostatic hyperplasia and prostatitis; (4) Participants with incomplete clinical information or who refused to provide serum samples. All study procedures were conducted in accordance with the Declaration of Helsinki and relevant institutional regulatory standards.

### Study design

**Figure 1:**
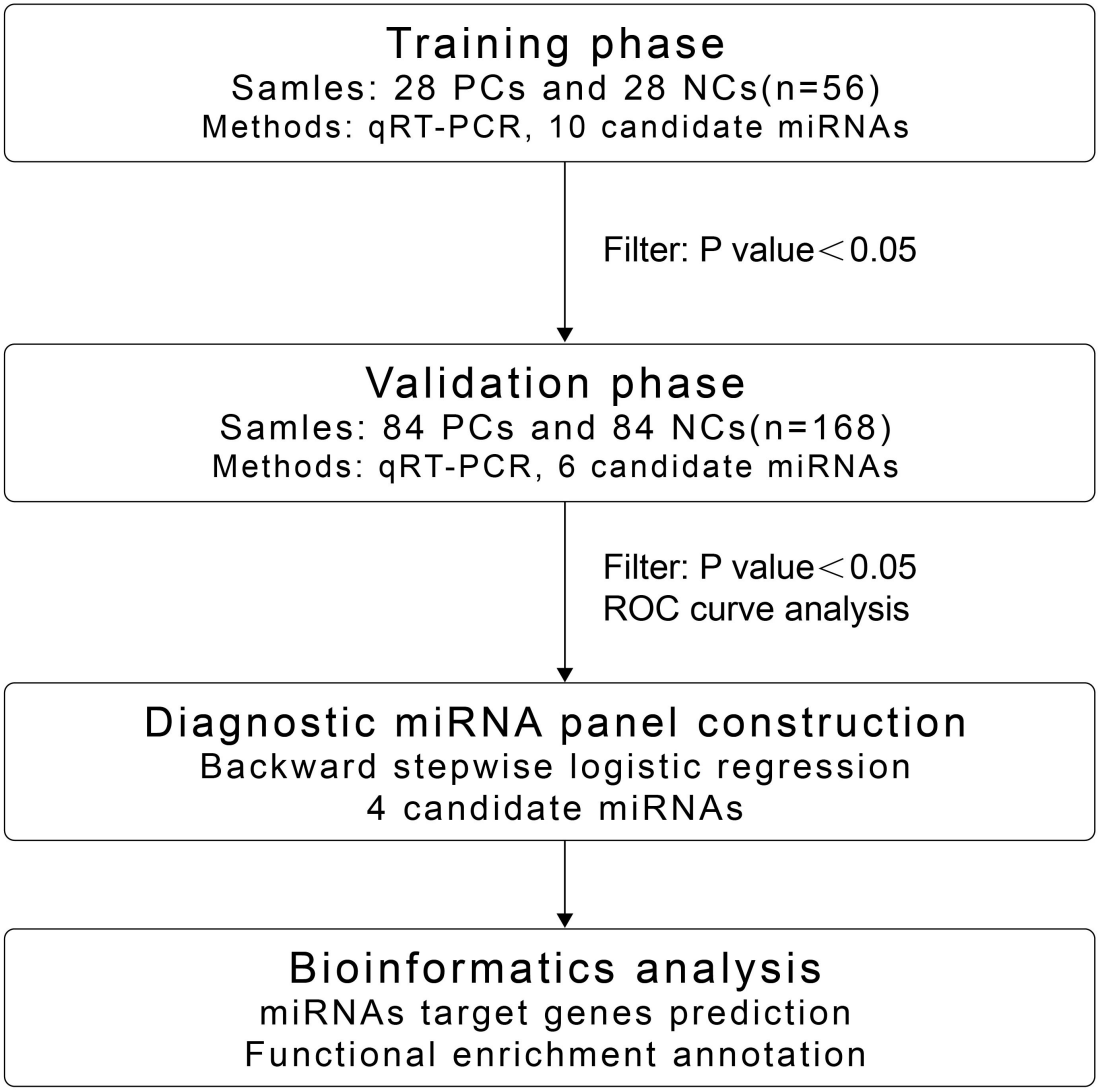
The experimental procedure of this study. PC, prostate cancer; NC, normal control; miRNA, microRNA; ROC, receiver operating characteristic; RT-qPCR, reverse transcription quantitative polymerase chain reaction.

**Figure 2:**
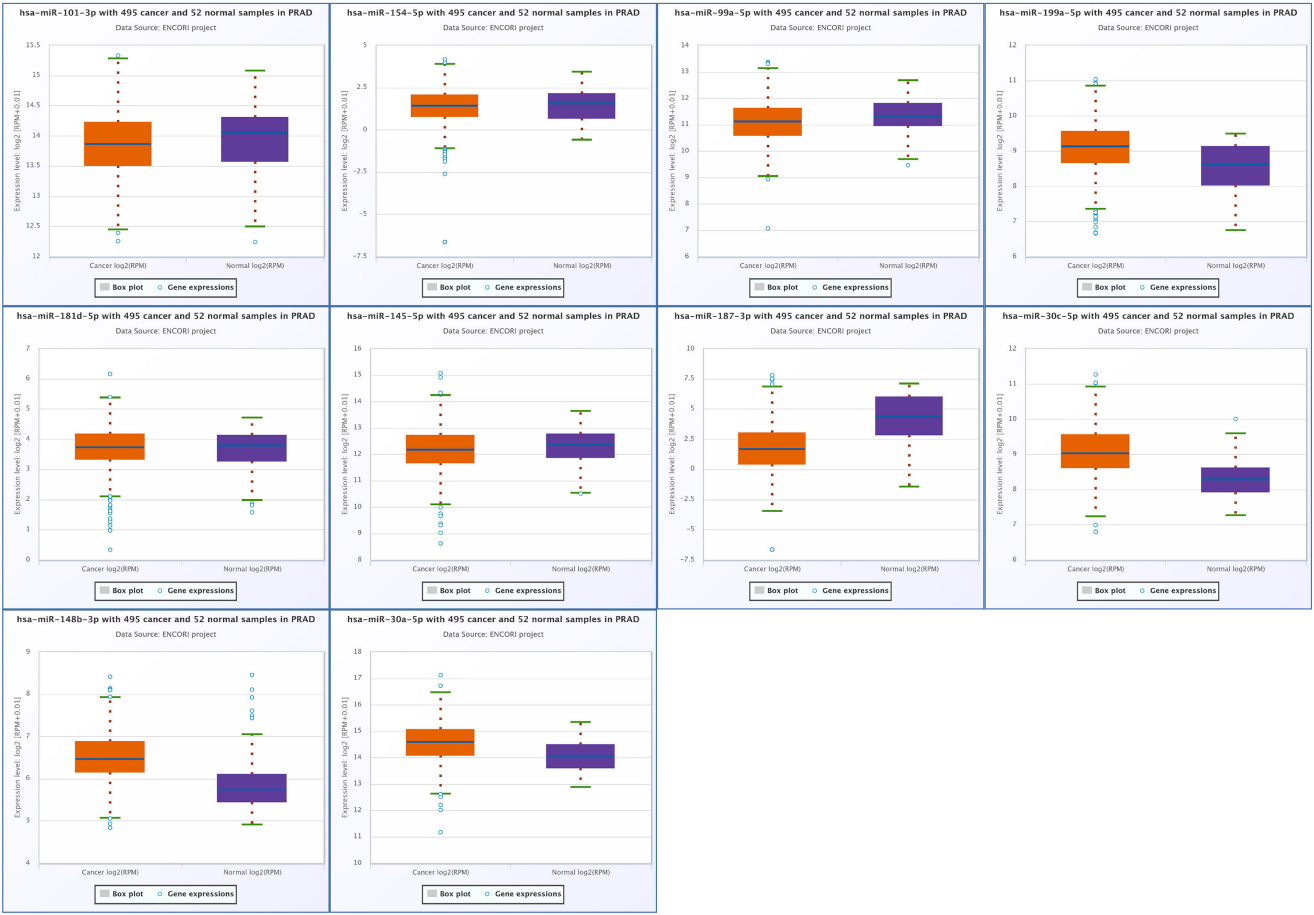
Expression of 10 PRAD-related miRNAs in ENCORI database. Box plots display the log_2_(RPM+0.01) expression levels of 10 miRNAs in 495 prostate cancer and 52 normal prostate tissues from the ENCORI project. Orange boxes: cancer tissues; purple boxes: normal tissues.

As shown in **Figure 1**, this study adopted a three-step design. First, we searched the PubMed database to screen miRNAs with aberrant expression in PC and regarded these molecules as candidate miRNAs. We then further screened these miRNAs via the Encyclopedia of RNA Interactions (ENCORI) database to obtain final candidate miRNAs (**Figure 2**). During the training phase, RT−qPCR was applied to detect the expression levels of these mature miRNAs in serum samples. We randomly examined miRNA expression in serum from 28 PC and 28 NC. With a *P*-value threshold set at less than 0.05, miRNAs with potential diagnostic efficacy were screened for subsequent validation. In the validation phase, we expanded the sample size and detected the expression of these miRNAs in serum samples from 84 PC and 84 NC. To further verify the diagnostic performance of these miRNAs, we calculated *P*-values, plotted ROC curves, and determined the corresponding AUC values. We explicitly state in the text that the samples of the training cohort and validation cohort were completely independent with no overlapping individuals. All participants were randomly recruited and assigned to different cohorts in a one-off manner, which avoids sample repetition and ensures the reliability of two-phase analysis results. Finally, backward stepwise logistic regression analysis was employed to identify a panel of mature miRNAs with optimal diagnostic performance. In addition, bioinformatics analysis was conducted on these mature miRNAs to further explore their biological functions and clinical implications.

### Serum sample collection and extraction of mature miRNAs

No anti−tumor treatment was administered to PC patients before sample collection. To reduce systematic variability during sample extraction, 2 μL of synthetic cel-miR-54-5p (10 nm/L, RiboBio, China) was added as an external reference. Subsequently, total RNA was isolated from serum samples using the TRIzol LS Reagent Kit (Thermo Fisher Scientific, Waltham, MA, USA). The concentration and purity of isolated total RNA were measured using a NanoDrop 2000c spectrophotometer (Thermo Scientific, USA).

### RT-qPCR section

RT−qPCR was performed to accurately quantify the expression of these mature miRNAs. Mature miRNAs were amplified using reverse transcription−specific primers from the Bulge-Loop miRNA RT-qPCR Primer Kit (RiboBio, Guangzhou, China). In addition, RT−qPCR reactions were carried out on a LightCycler 480 real−time PCR system (Roche Diagnostics, Mannheim, Germany) with TaqMan probes. RT−qPCR was run for 40 cycles under the following conditions: initial pre−denaturation at 95 °C for 20 s, followed by cyclic steps of denaturation at 95 °C for 10 s, annealing at 60 °C for 20 s, and extension at 70 °C for 10 s. Finally, the 2^-Δ^*^Cq^* method was adopted to quantify the relative expression of candidate mature miRNAs for intergroup comparison.

### Bioinformatics analysis

Target genes of candidate miRNAs were screened via the miRWalk 3.0 database (29). The GEPIA database (30) was used to further verify the correlation between predicted target genes and PC. Using the DAVID database (31, 32), we conducted Gene Ontology (GO) functional annotation and pathway analysis on common target genes predicted by two or more miRNAs via an online bioinformatics platform, and visualized the analytical results (33).

### Statistical analysis

The 2^-ΔΔ^*^Cq^* method was applied to calculate the relative expression levels of miRNAs (34). Categorical variables among groups were presented as counts and percentages, whereas continuous variables were expressed as mean ± standard deviation. The Kruskal-Wallis rank-sum test was adopted for comparisons among multiple independent groups. Differences in the expression of candidate miRNAs between PC and HC samples were analyzed using the Student’s *t*−test or the Mann−Whitney *U* test. Multiple logistic regression analysis was used to construct diagnostic miRNA panels. ROC curves and corresponding AUC values were analyzed to evaluate the diagnostic efficacy of these miRNAs. A *P*-value < 0.05 was defined as statistically significant. All statistical analyses were conducted using SPSS 26.0 (SPSS Inc., Chicago, IL, USA), GraphPad Prism 9.0 (version 9.4.1.681), and MedCalc 19.

## Results

### Participant characteristics

This study included a total of 224 participants, with 112 cases in the PC group and 112 cases in the NC group. The demographic characteristics of all participants are presented in **Table 1**. PC was confirmed by postoperative pathological biopsy. All participants did not receive any treatment before serum collection and had no history of other malignant tumors. Participants in the PC group received no treatment before blood collection. The PC group was stratified by age and matched with the NC group. The NC group consisted of healthy volunteers who received routine physical examination and had no history of malignant diseases. **Table 1** summarizes the baseline demographic characteristics and clinical data of the 224 participants in the training and validation cohorts. There was no statistically significant difference in age distribution between the PC and NC groups (*P* > 0.05).

**Table 1:** Outcomes of receiver operating characteristic curves for different panels.

	AUC (95% CI)	P value	Youden index	Cut-off	Sensitivity	Specificity
5-miRNA panel	0.850 (0.787 to 0.901)	< 0.0001	0.5833	>0.5129	79.76%	78.57%
Best of 4-miRNA panels						
Panel 1	0.825 (0.759 to 0.879)	< 0.0001	0.5238	>0.4332	83.33%	69.05%
Panel 2	0.836 (0.771 to 0.889)	< 0.0001	0.6071	>0.5226	82.14%	78.57%
Panel 3	0.791 (0.721 to 0.849)	< 0.0001	0.5000	> 0.5411	72.62%	77.38%
Panel 4	0.819 (0.752 to 0.874)	< 0.0001	0.5476	>0.5627	72.62%	82.14%
Panel 5	0.832 (0.767 to 0.885)	< 0.0001	0.5476	>0.6006	69.05%	85.71%
Best of 3-miRNA panels						
Panel 1	0.771 (0.700 to 0.832)	< 0.0001	0.4524	>0.5266	70.24%	70.24%
Panel 2	0.805 (0.737 to 0.862)	< 0.0001	0.5119	>0.5432	72.62%	78.57%
Panel 3	0.770 (0.699 to 0.831)	< 0.0001	0.4286	>0.6152	55.95%	86.90%
Panel 4	0.807 (0.739 to 0.864)	< 0.0001	0.5119	>0.5280	73.81%	77.38%
Panel 5	0.757 (0.685 to 0.820)	< 0.0001	0.3929	>0.5215	69.05%	70.24%
Panel 6	0.803 (0.735 to 0.860)	< 0.0001	0.4643	>0.5493	67.86%	78.57%
Panel 7	0.793 (0.724 to 0.852)	< 0.0001	0.4643	>0.5056	73.81%	72.62%
Panel 8	0.775 (0.704 to 0.836)	< 0.0001	0.4643	>0.5416	70.24%	76.19%
Panel 9	0.774 (0.703 to 0.835)	< 0.0001	0.4524	>0.5317	67.86%	77.38%
Panel 10	0.799 (0.731 to 0.857)	< 0.0001	0.4762	>0.4926	76.19%	71.43%

AUC, area under curve; CI, confidence interval.

### Screening of candidate miRNAs

First, we searched PubMed for studies related to PC and mature miRNAs, and screened mature miR molecules with aberrant expression in PC. We then used the ENCORI database to further screen candidate miRNAs according to expression levels. The results showed significant expression differences among the following 10 candidate miRNAs: miR-101-3p, miR-154-5p, miR-99a-5p, miR-199a-5p, miR-181d-5p, miR-145-5p, miR-187-3p, miR-30c-5p, miR-148b-3p, and miR-30a-5p (**Figure 2**). Furthermore, accumulating studies have demonstrated that specific miRNAs, including miR-145-5p and miR-30a-5p, can inhibit the proliferation and metastasis of PC cells (35, 36), and are closely correlated with PC prognosis (37). However, there remains a paucity of literature regarding the specificity of their serum expression in PC. Thus, these miRNAs are considered promising candidate molecules worthy of further investigation.

### miRNA expression profiles during the training phase

**Figure 3:**
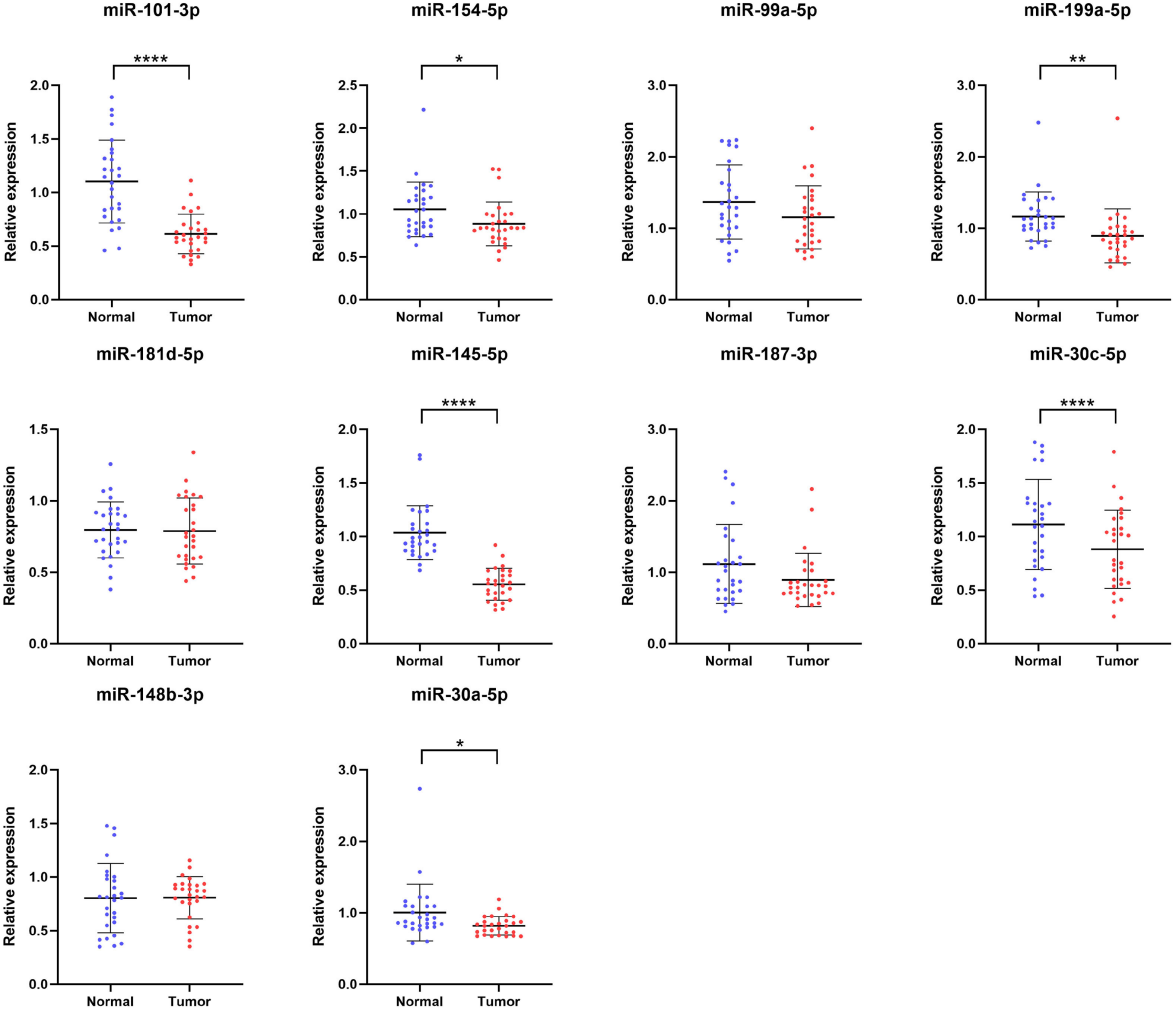
The relative expression of 10 candidate mature miRNAs in the training phase. * Denotes *p* < 0.05, ** denotes *p* < 0.01, *** denotes p < 0.001. This phase used 28 sera from PC patients and 28 healthy control sera.

During the training phase, RT−qPCR was applied to detect the serum expression levels of the 10 candidate miRNAs in 28 PC and 28 NC. As shown in **Figure 3**, six miRNAs (miR-101-3p, miR-154-5p, miR-199a-5p, miR-145-5p, miR-30c-5p, and miR-30a-5p) showed significantly differential expression (*P* < 0.05). Accordingly, we performed further experimental validation on these six candidate miRNAs.

### Diagnostic performance of key miRNAs during the validation phase

**Figure 4:**
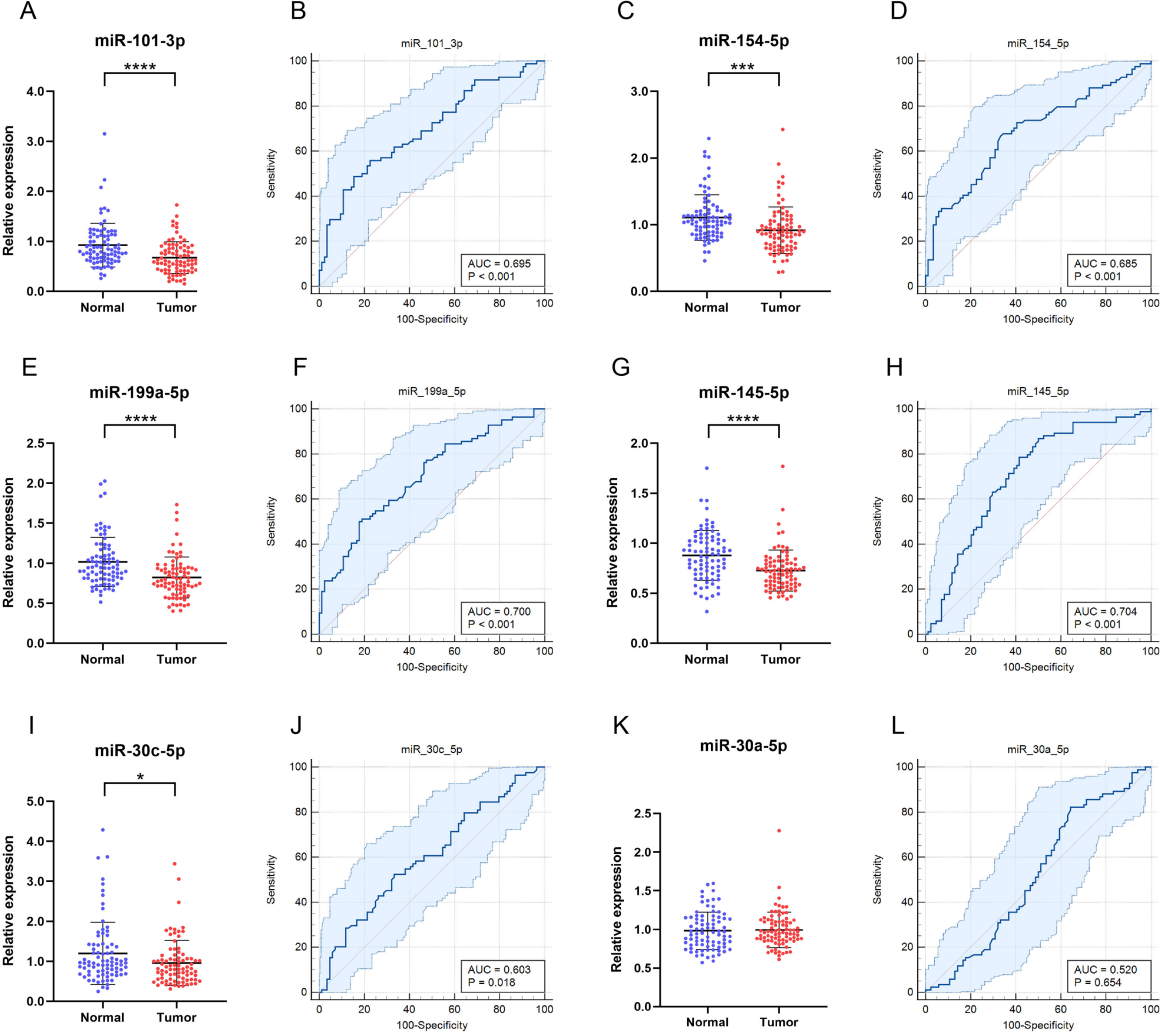
Expression levels of six mature miRNAs in serum and their corresponding ROC curves in the verification phase. This verification phase enrolled 84 PC sera and 84 NC sera. The expression levels of **(A, B)** miR-101-3p, **(C, D)** miR-154-5p, **(E, F)** miR-199a-5p, **(G, H)** miR-145-5p, and **(I, J)** miR-30c-5p in PC sera were significantly downregulated compared with those in NC; the expression of **(K, L**) miR-30a-5p showed no significant difference (p = 0.64). Statistical significance: *p < 0.05, **p < 0.01, ***p < 0.001. For ROC curve analyses, the red solid line represents the ROC curve, the green shaded area represents the 95% confidence interval, and the blue line represents the diagonal reference line. (For interpretation of the reference to color in this figure legend, the reader is referred to the web version of this article.).

During the validation phase, RT−qPCR was used to detect the expression levels of these six key miRNAs in serum samples from 84 PC and 84 NC. To evaluate the diagnostic efficacy of the key miRNAs, ROC curves were constructed, and corresponding AUC values and Youden’s index were calculated. The diagnostic results of these six key miRNAs are presented in **Figure 4** and **Table 2**. Five of these key miRNAs still presented significantly differential expression and favorable diagnostic performance. The AUC and corresponding 95% CIs were as follows:miR-101-3p: 0.695 (95% CI: 0.619–0.763; **Figure 4B**); miR-154-5p: 0.685 (95% CI: 0.609–0.754; **Figure 4D**); miR-199a-5p: 0.700 (95% CI: 0.624–0.768; **Figure 4F**);miR-145-5p: 0.704 (95% CI: 0.629–0.772; **Figure 4H**); miR-30c-5p: 0.603 (95% CI: 0.525–0.678; **Figure 4J**). Furthermore, we calculated the Youden’s index, sensitivity and specificity of the above miRNAs for PC diagnosis, with detailed values summarized in **Table 2**, to comprehensively assess their diagnostic performance.

**Table 2:** Comparison of diagnostic performance of the 4-miRNA signature in different validation scenarios.

Sample ID	Group Label	miR-101-3p	miR-154-5p	miR-199a-5p	miR-145-5p
Normal Group1	Normal	0.2325	-0.38	-0.475	0.2125
Normal Group2	Normal	0.6275	-0.425	-1.31	0.0775
Normal Group3	Normal	0.0625	-0.555	-0.555	0.2625
Normal Group4	Normal	-0.3975	-0.28	-0.5	0.0975
Normal Group5	Normal	0.4675	0.325	-0.075	-0.0225
Normal Group6	Normal	0.1625	-0.16	-0.51	0.1175
Normal Group7	Normal	-0.2725	-0.05	-0.35	0.4425
Normal Group8	Normal	-0.7125	0.21	-0.245	0.5475
Normal Group9	Normal	-0.7825	-0.35	-0.315	0.2725
Normal Group10	Normal	-0.4525	0.105	-0.175	-0.0775
Normal Group11	Normal	-0.2825	-0.215	0.47	-0.1625
Normal Group12	Normal	-0.9175	0.4	-0.095	-0.3575
Normal Group13	Normal	-0.0425	0.655	0.29	-0.0675
Normal Group14	Normal	0.2575	0.105	-0.185	0.0175
Normal Group15	Normal	0.585	-0.0725	-0.01	-0.3
Normal Group16	Normal	-0.825	-0.4075	-0.68	-0.1
Normal Group17	Normal	0.43	0.2925	0.035	-0.155
Normal Group18	Normal	0.235	-0.2525	-0.19	-0.31
Normal Group19	Normal	-0.385	-0.2275	-0.015	0.105
Normal Group20	Normal	-0.575	0.1625	0.05	0.21
Normal Group21	Normal	-0.195	0.2275	-0.045	0.01
Normal Group22	Normal	-0.29	0.2325	-0.49	0.305
Normal Group23	Normal	1.065	-1.1475	0.01	0.135
Normal Group24	Normal	1.125	0.4475	-0.21	-0.815
Normal Group25	Normal	0.37	-0.2025	0.28	-0.785
Normal Group26	Normal	-0.49	0.3725	0.41	-0.32
Normal Group27	Normal	-0.21	0.4325	-0.085	0.075
Normal Group28	Normal	-0.125	0.1575	0.325	0.205
Prostate Cancer1	Prostate Cancer	0.8975	0.5725	-0.0375	0.655
Prostate Cancer2	Prostate Cancer	0.6225	-0.0025	0.4675	0.745
Prostate Cancer3	Prostate Cancer	0.6625	-0.6075	0.3225	0.56
Prostate Cancer4	Prostate Cancer	0.7775	-0.6025	0.0575	1.255
Prostate Cancer5	Prostate Cancer	0.2775	0.2875	0.2325	1.365
Prostate Cancer6	Prostate Cancer	1.1375	0.2975	0.3075	0.965
Prostate Cancer7	Prostate Cancer	0.9025	0.0075	0.8825	0.545
Prostate Cancer8	Prostate Cancer	1.4475	1.1125	0.5125	1.625
Prostate Cancer9	Prostate Cancer	0.8475	0.2625	0.9925	1.665
Prostate Cancer10	Prostate Cancer	0.6925	0.0275	0.8625	1.475
Prostate Cancer11	Prostate Cancer	1.2675	-0.5075	0.4125	0.76
Prostate Cancer12	Prostate Cancer	0.7275	0.4825	0.7825	0.65
Prostate Cancer13	Prostate Cancer	1.5975	0.1725	0.7375	0.285
Prostate Cancer14	Prostate Cancer	-0.1525	0.7225	0.3275	0.5
Prostate Cancer15	Prostate Cancer	0.2225	0.3125	-0.1975	0.555
Prostate Cancer16	Prostate Cancer	0.0275	0.2575	-0.0075	0.465
Prostate Cancer17	Prostate Cancer	0.4825	0.2525	-0.2575	1.005
Prostate Cancer18	Prostate Cancer	0.5875	0.1325	-0.1825	0.89
Prostate Cancer19	Prostate Cancer	0.9375	0.0025	0.2225	1.11
Prostate Cancer20	Prostate Cancer	0.6125	0.4575	0.1025	0.835
Prostate Cancer21	Prostate Cancer	0.5125	0.4975	-0.0275	1.075
Prostate Cancer22	Prostate Cancer	0.2225	0.2525	-1.3425	0.81
Prostate Cancer23	Prostate Cancer	0.7925	0.1325	0.1375	1.405
Prostate Cancer24	Prostate Cancer	0.8025	0.2625	0.0775	1.085
Prostate Cancer25	Prostate Cancer	1.3125	0.6225	0.1425	0.86
Prostate Cancer26	Prostate Cancer	1.1075	-0.0975	0.2575	0.77
Prostate Cancer27	Prostate Cancer	1.3225	0.8225	1.1275	1.3
Prostate Cancer28	Prostate Cancer	0.8225	0.3225	0.1375	0.12
Normal Group29	Normal	-0.6925	-0.2025	-0.0325	0.47
Normal Group30	Normal	-0.7175	-0.5275	-0.5225	0.28
Normal Group31	Normal	0.5575	-0.3725	-0.9925	0.53
Normal Group32	Normal	-0.1825	-0.0375	-0.8775	0.64
Normal Group33	Normal	-0.1275	0.3675	-0.0675	0.41
Normal Group34	Normal	-0.7325	-0.0675	-0.5625	1.085
Normal Group35	Normal	-0.4925	0.2425	-0.1225	1.095
Normal Group36	Normal	-0.1225	0.0475	-0.5425	0.845
Normal Group37	Normal	-0.3175	0.4125	0.1525	0.865
Normal Group38	Normal	-0.4325	0.2025	0.0725	0.125
Normal Group39	Normal	0.5575	0.5275	0.4775	0.63
Normal Group40	Normal	0.3725	-0.7575	-0.2475	0.565
Normal Group41	Normal	0.1075	0.3225	-0.5825	0.405
Normal Group42	Normal	-0.2875	-0.6525	-0.0225	0.295
Normal Group43	Normal	-1.1575	-0.48	-0.235	-0.0325
Normal Group44	Normal	0.3425	-0.29	0.3	0.4275
Normal Group45	Normal	0.2275	-1.01	0.45	0.1725
Normal Group46	Normal	-0.3125	-0.34	0.275	0.1025
Normal Group47	Normal	-0.2675	0.14	0.195	-0.1325
Normal Group48	Normal	0.6525	-1.065	0.005	-0.2325
Normal Group49	Normal	0.8325	-0.2	0.47	-0.0725
Normal Group50	Normal	0.0075	-0.665	0.345	0.4675
Normal Group51	Normal	0.9975	-0.145	0.74	0.0925
Normal Group52	Normal	1.0525	-0.885	0.36	0.1125
Normal Group53	Normal	0.1125	-0.015	0.6	0.2525
Normal Group54	Normal	0.1575	0.005	0.32	-0.0525
Normal Group55	Normal	-0.1875	0.765	-0.29	-0.5125
Normal Group56	Normal	0.4325	-0.63	0.57	-0.8075
Normal Group57	Normal	0.02	0.4675	0.3125	0.11
Normal Group58	Normal	0.105	0.1725	-0.0175	0.03
Normal Group59	Normal	0.78	0.2925	0.0825	0.22
Normal Group60	Normal	0.605	-0.2675	0.0775	0.205
Normal Group61	Normal	0.735	-0.1175	0.3025	0.445
Normal Group62	Normal	0.405	-0.1575	0.0325	0.265
Normal Group63	Normal	-0.27	-0.4325	-0.0925	0.35
Normal Group64	Normal	1.1	0.1175	0.2175	0.66
Normal Group65	Normal	-0.175	-0.2675	0.1725	0.72
Normal Group66	Normal	0.245	-0.2775	0.4425	0.8
Normal Group67	Normal	0.625	0.2875	0.3475	0.335
Normal Group68	Normal	0.595	-1.1975	0.2175	-0.095
Normal Group69	Normal	0.34	-0.1775	0.3375	0.535
Normal Group70	Normal	-0.05	0.3825	0.1675	0.165
Normal Group71	Normal	0.185	1.1275	-0.495	0.4075
Normal Group72	Normal	-0.17	0.2275	-0.14	-0.0375
Normal Group73	Normal	-0.255	0.2675	-0.535	-0.0125
Normal Group74	Normal	-1.055	0.0575	0	0.5225
Normal Group75	Normal	0.005	-0.0725	-0.905	0.6175
Normal Group76	Normal	0.83	0.2575	0.085	1.0025
Normal Group77	Normal	-0.65	-0.4225	-0.3	0.3875
Normal Group78	Normal	0.44	-0.1025	-0.31	0.7575
Normal Group79	Normal	0.68	-0.0325	0.62	0.6575
Normal Group80	Normal	-0.225	-0.1725	0.96	1.1575
Normal Group81	Normal	0.275	0.1825	-0.175	1.0225
Normal Group82	Normal	0.385	0.0325	0.17	1.6625
Normal Group83	Normal	1.075	-0.2275	-0.445	0.8425
Normal Group84	Normal	0.805	-0.0725	-0.13	0.3825
Normal Group85	Normal	1.5425	-0.05	0.095	-0.3
Normal Group86	Normal	0.3275	-0.14	0.26	-0.515
Normal Group87	Normal	0.4225	-1.02	-0.055	-0.27
Normal Group88	Normal	0.7325	-0.33	0.15	0
Normal Group89	Normal	0.4525	-0.26	-0.03	0.075
Normal Group90	Normal	0.3825	0.225	0.3	-0.125
Normal Group91	Normal	-0.1475	-0.065	0.43	0.07
Normal Group92	Normal	0.3425	0.765	0.555	-0.09
Normal Group93	Normal	0.4075	-0.79	0.045	0.125
Normal Group94	Normal	0.7175	0.2	0.48	0.125
Normal Group95	Normal	0.4125	0.4	0.625	-0.185
Normal Group96	Normal	1.9475	-0.195	0.565	-0.04
Normal Group97	Normal	1.6475	-0.07	0.515	-0.43
Normal Group98	Normal	0.1825	-0.055	0.155	-0.22
Normal Group99	Normal	0.185	0.305	0.11	-0.2
Normal Group100	Normal	-1.655	-0.095	-0.39	-0.25
Normal Group101	Normal	-0.305	0.04	-0.5	-0.03
Normal Group102	Normal	0.745	-0.045	-0.31	-0.155
Normal Group103	Normal	0.565	-0.155	-0.075	0.13
Normal Group104	Normal	0.55	0.345	-0.08	0.28
Normal Group105	Normal	-0.14	-0.175	-0.45	-0.125
Normal Group106	Normal	1.085	0.045	-1.02	0.005
Normal Group107	Normal	0.18	-0.11	0.22	0.32
Normal Group108	Normal	-0.275	0.165	-0.225	-0.075
Normal Group109	Normal	0.275	-0.58	-0.105	0.11
Normal Group110	Normal	0.485	0.01	0.09	0.33
Normal Group111	Normal	1.13	-0.185	0.19	0.275
Normal Group112	Normal	0.71	0.095	0.21	-0.165
Prostate Cancer29	Prostate Cancer	0.75	0.5525	0.2525	0.285
Prostate Cancer30	Prostate Cancer	1.18	0.2325	0.0425	0.435
Prostate Cancer31	Prostate Cancer	0.49	0.0575	-0.3025	0.485
Prostate Cancer32	Prostate Cancer	0.305	0.1825	-0.1925	0.135
Prostate Cancer33	Prostate Cancer	1.105	0.3275	0.0925	0.64
Prostate Cancer34	Prostate Cancer	0.705	0.1875	0.0225	0.195
Prostate Cancer35	Prostate Cancer	1.3	0.2525	0.4925	1.08
Prostate Cancer36	Prostate Cancer	0.19	0.6875	0.4325	0.42
Prostate Cancer37	Prostate Cancer	0.21	-0.3275	-0.1875	0.85
Prostate Cancer38	Prostate Cancer	0.145	-0.5275	0.4225	1.11
Prostate Cancer39	Prostate Cancer	0.105	-0.1825	0.4525	0.21
Prostate Cancer40	Prostate Cancer	0.705	0.5825	0.4825	0.98
Prostate Cancer41	Prostate Cancer	1.295	0.6125	0.8325	0.23
Prostate Cancer42	Prostate Cancer	0.365	0.0275	0.0975	0.065
Prostate Cancer43	Prostate Cancer	0.8925	0.8325	0.19	0.3225
Prostate Cancer44	Prostate Cancer	1.5575	0.0075	0.24	-0.1525
Prostate Cancer45	Prostate Cancer	0.9075	0.1125	0.105	0.4275
Prostate Cancer46	Prostate Cancer	1.2475	0.1575	0.375	1.0975
Prostate Cancer47	Prostate Cancer	1.6825	0.1825	0.155	0.9275
Prostate Cancer48	Prostate Cancer	1.0025	0.0475	0.325	0.6125
Prostate Cancer49	Prostate Cancer	1.3925	0.0025	0.315	0.1225
Prostate Cancer50	Prostate Cancer	0.8425	0.2925	0.4	1.1775
Prostate Cancer51	Prostate Cancer	2.2925	0.2825	0.4	0.0425
Prostate Cancer52	Prostate Cancer	2.2575	0.6425	0.685	0.8375
Prostate Cancer53	Prostate Cancer	2.7525	1.8075	0.42	0.4775
Prostate Cancer54	Prostate Cancer	0.7675	0.5225	0.835	0.4575
Prostate Cancer55	Prostate Cancer	1.1125	0.7375	0.295	-0.1375
Prostate Cancer56	Prostate Cancer	-0.0375	0.6275	0.415	0.5125
Prostate Cancer57	Prostate Cancer	0.1225	0.875	0.35	0.465
Prostate Cancer58	Prostate Cancer	0.1275	0.72	0.565	0.375
Prostate Cancer59	Prostate Cancer	0.5225	-0.46	0.305	0.595
Prostate Cancer60	Prostate Cancer	0.6975	0.105	0.015	0.205
Prostate Cancer61	Prostate Cancer	0.4075	1.15	-0.16	0.655
Prostate Cancer62	Prostate Cancer	-0.0625	0.625	0.505	0.44
Prostate Cancer63	Prostate Cancer	1.5175	0.15	0.18	0.72
Prostate Cancer64	Prostate Cancer	0.4275	0.42	0.565	0.435
Prostate Cancer65	Prostate Cancer	1.2175	-0.405	0.1	0.595
Prostate Cancer66	Prostate Cancer	-0.3575	-0.11	1.05	0.8
Prostate Cancer67	Prostate Cancer	-0.7875	-0.78	0.38	0.575
Prostate Cancer68	Prostate Cancer	0.6725	-0.085	-0.625	0.05
Prostate Cancer69	Prostate Cancer	-0.2425	0.695	0.47	1.14
Prostate Cancer70	Prostate Cancer	-0.3925	1.115	0.075	0.27
Prostate Cancer71	Prostate Cancer	2.34	0.4075	0.4725	0.2825
Prostate Cancer72	Prostate Cancer	0.845	0.2175	0.4925	0.3275
Prostate Cancer73	Prostate Cancer	0.67	-0.9325	0.7575	0.7975
Prostate Cancer74	Prostate Cancer	1.355	0.4725	0.4675	0.7175
Prostate Cancer75	Prostate Cancer	0.895	0.1075	0.7375	0.9175
Prostate Cancer76	Prostate Cancer	0.59	0.5725	0.6375	0.9875
Prostate Cancer77	Prostate Cancer	1.88	0.1575	0.5425	-0.2525
Prostate Cancer78	Prostate Cancer	0.765	-0.0975	1.0525	0.3775
Prostate Cancer79	Prostate Cancer	0.035	0.0575	0.8075	-0.4175
Prostate Cancer80	Prostate Cancer	0.455	0.9025	1.0825	0.6225
Prostate Cancer81	Prostate Cancer	1.66	0.0625	0.5025	-0.8225
Prostate Cancer82	Prostate Cancer	1.475	0.2125	1.3175	0.2475
Prostate Cancer83	Prostate Cancer	1.28	-0.0375	1.1475	0.4975
Prostate Cancer84	Prostate Cancer	0.11	0.4525	0.7675	0.7725
Prostate Cancer85	Prostate Cancer	0.7675	0.3225	-0.3075	0.2825
Prostate Cancer86	Prostate Cancer	2.0675	-0.7125	-0.7925	0.3075
Prostate Cancer87	Prostate Cancer	0.9475	-0.2225	1.2925	0.3775
Prostate Cancer88	Prostate Cancer	-0.0375	-0.0725	-0.7075	0.6925
Prostate Cancer89	Prostate Cancer	-0.5875	-0.2475	-0.4475	0.5375
Prostate Cancer90	Prostate Cancer	0.3925	0.4825	-0.0375	0.4075
Prostate Cancer91	Prostate Cancer	0.3575	0.2325	0.1675	0.4375
Prostate Cancer92	Prostate Cancer	0.0575	0.0875	0.8475	0.4025
Prostate Cancer93	Prostate Cancer	0.7275	0.8375	1.0525	0.3475
Prostate Cancer94	Prostate Cancer	0.8675	0.1575	0.1825	0.2575
Prostate Cancer95	Prostate Cancer	0.9175	-0.6425	0.0825	0.5475
Prostate Cancer96	Prostate Cancer	-0.4425	-0.1175	0.4725	0.4325
Prostate Cancer97	Prostate Cancer	-0.1175	-0.2575	0.2775	0.5325
Prostate Cancer98	Prostate Cancer	1.2675	1.7775	0.5825	0.0675
Prostate Cancer99	Prostate Cancer	0.795	-0.185	0.11	0.8175
Prostate Cancer100	Prostate Cancer	-0.485	0.845	0.135	0.8275
Prostate Cancer101	Prostate Cancer	0.3	-0.695	-0.09	0.8975
Prostate Cancer102	Prostate Cancer	1	0.29	0.025	0.9725
Prostate Cancer103	Prostate Cancer	0.315	1.165	0.21	0.7375
Prostate Cancer104	Prostate Cancer	0.23	0.19	-0.12	0.8725
Prostate Cancer105	Prostate Cancer	1.625	-0.01	0.83	0.9425
Prostate Cancer106	Prostate Cancer	0.835	-0.18	-0.185	0.8625
Prostate Cancer107	Prostate Cancer	0.475	0.08	0.105	0.7425
Prostate Cancer108	Prostate Cancer	0.195	-0.255	0.19	0.7725
Prostate Cancer109	Prostate Cancer	2.075	0.69	1.095	0.7625
Prostate Cancer110	Prostate Cancer	1.235	-1.28	0.125	0.6825
Prostate Cancer111	Prostate Cancer	0.09	0.55	0.885	0.6875
Prostate Cancer112	Prostate Cancer	0.345	0.24	0.71	0.3375

### Identify the most suitable miRNA combination

**Figure 5:**
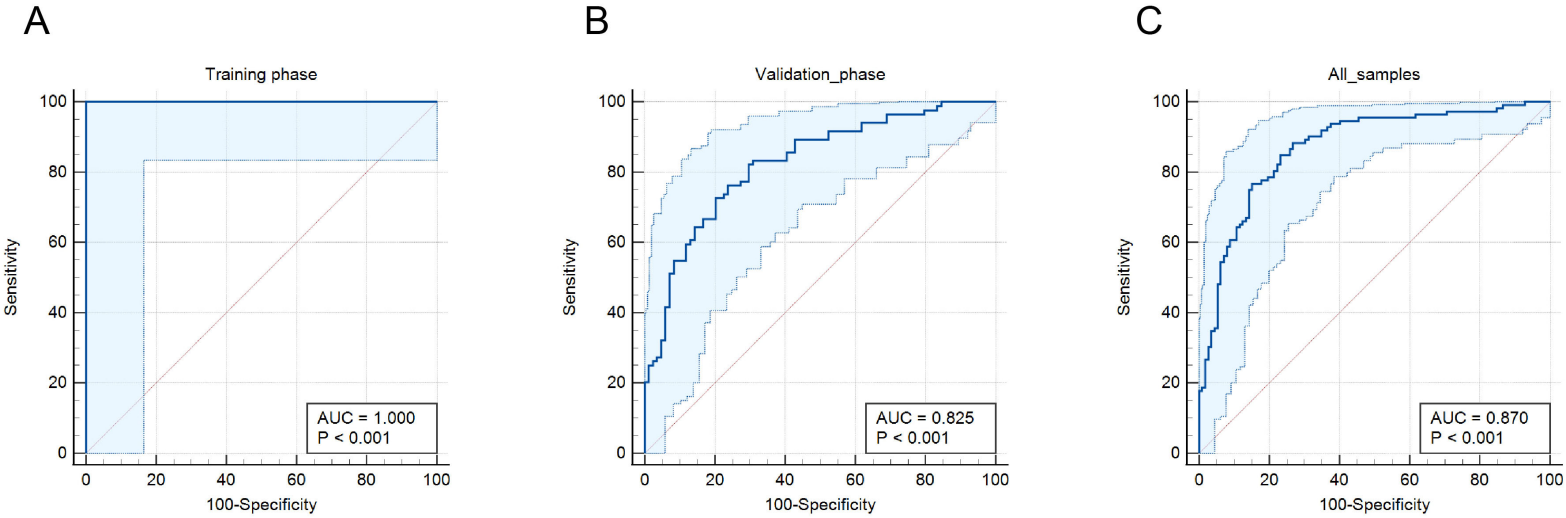
Analysis of the ROC curves for the 4-miRNA panel. **(A)** Training phase, **(B)** Validation phase, **(C)** All samples. The dark blue solid line represents the ROC curve of the 4-miRNA signature, the light blue shaded area represents the 95% confidence interval (CI), and the red solid line indicates the diagonal reference line. (For interpretation of the reference to color in this figure legend, the reader is referred to the web version of this article).

Combinations of multiple miRNAs may yield superior diagnostic performance compared with individual miRNAs, but they remarkably increase the cost of post−transcriptional detection. To screen optimal miRNA combinations that balance diagnostic efficacy and detection cost, we systematically evaluated the diagnostic performance of a 5−miRNA panel (1 model), five 4−miRNA panels (5 models), and ten 3−miRNA panels (10 models). **Table 3** presents the optimal panels for miRNA combinations with different quantities. After balancing diagnostic efficacy and testing costs, we finally selected the optimal four-miRNA panel consisting of miR-101-3p, miR-154-5p, miR-199a-5p, and miR-145-5p as the ultimate diagnostic biomarker panel for PC in this study. This four−miRNA panel exhibited favorable diagnostic efficacy, with an AUC of 0.825, a sensitivity of 83.33%, and a specificity of 69.05%. The logistic regression equation of this model was as follows: Logit(p) = 7.782 − 2.169 × miR-101-3p − 1.372 × miR-154-5p − 1.989 × miR-199a-5p − 3.635 × miR-145-5p. The AUC values of this four−miRNA panel were 0.825, 1.000 and 0.870 for the validation set, test set and overall dataset, respectively (**Figure 5**).

**Table 3:** Outcomes of receiver operating characteristic curves for different panels.

	AUC (95% CI)	P value	Youden index	Cut-off	Sensitivity	Specificity
5-miRNA panel	0.850 (0.787 to 0.901)	< 0.0001	0.5833	>0.5129	79.76%	78.57%
Best of 4-miRNA panels	0.825 (0.759 to 0.879)	< 0.0001	0.5238	>0.4332	83.33%	69.05%
Best of 3-miRNA panels	0.807 (0.739 to 0.864)	< 0.0001	0.5119	>0.5280	73.81%	77.38%

AUC, area under curve; CI, confidence interval.

### Bioinformatics analysis of candidate miRNAs

**Figure 6:**
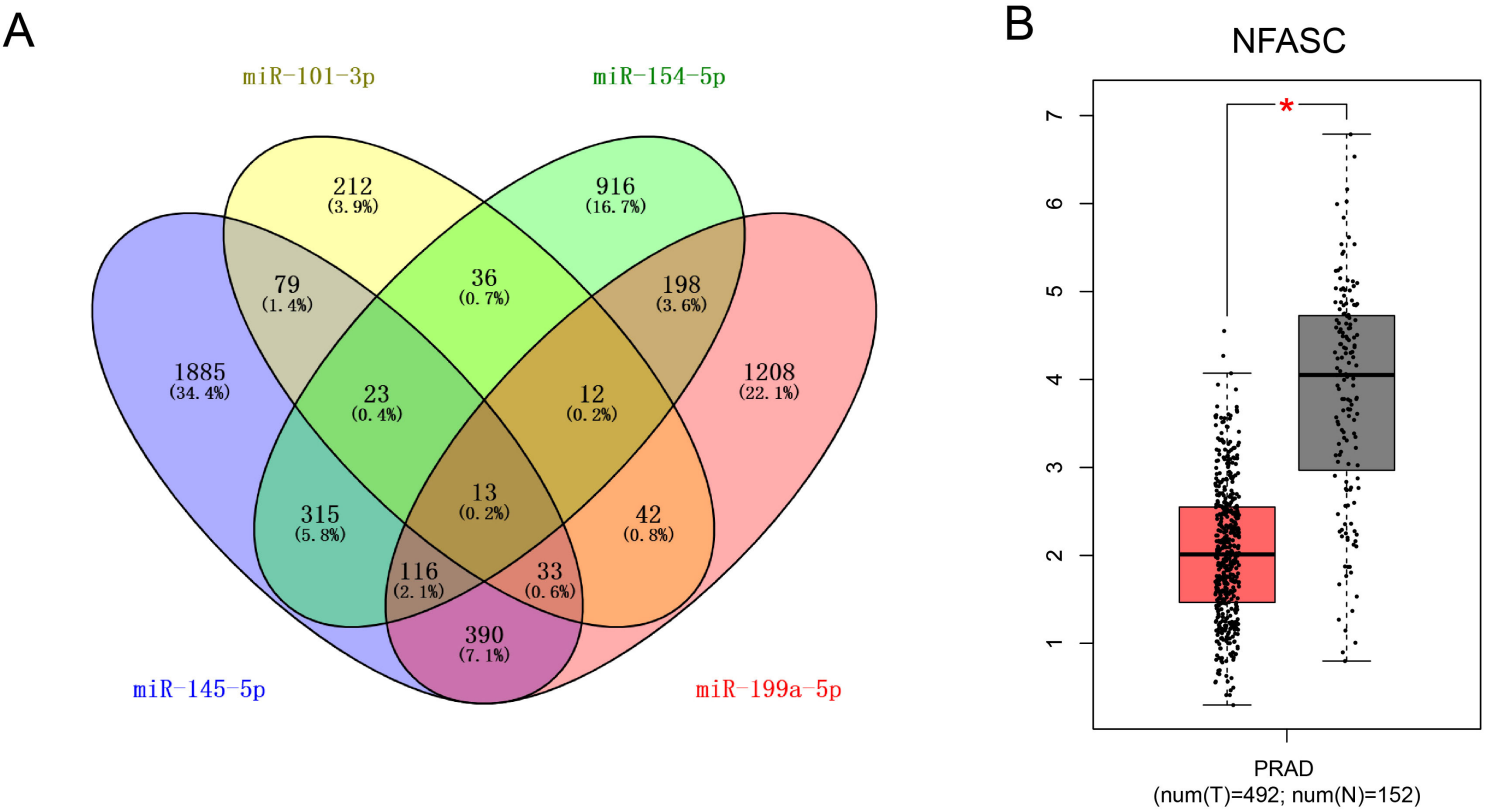
Target gene prediction and differentially expressed gene analysis in GEPIA database. **(A)** Venn diagram of common target genes for the four miRNAs (miR-101-3p, miR-154-5p, miR-145-5p, miR-199a-5p) predicted by integrated bioinformatics databases. **(B)** Expression of *NFASC* was significantly dysregulated in 492 PRAD patients vs. 152 normal controls, with |log_2_FC| > 0.1 and *p* < 0.05.

To further explore the potential molecular mechanisms and biological functions of these four key miRNAs in PC, miRWalk 3.0 was used to predict their downstream target genes. The predicted target genes of the four miRNAs were visualized via a Venn diagram (**Figure 6A**). A total of 13 target genes were co−predicted by all four key miRNAs. Expression analysis of these 13 genes was performed using the GEPIA platform, which integrates transcriptome data from the TCGA and GTEx databases. In PC, the *NFASC* gene showed significantly differential expression (|log_2_ fold change| > 1.0, *P* < 0.01; **Figure 6B**). Thus, *NFASC* may serve as a critical common target of the four key miRNAs.

**Figure 7:**
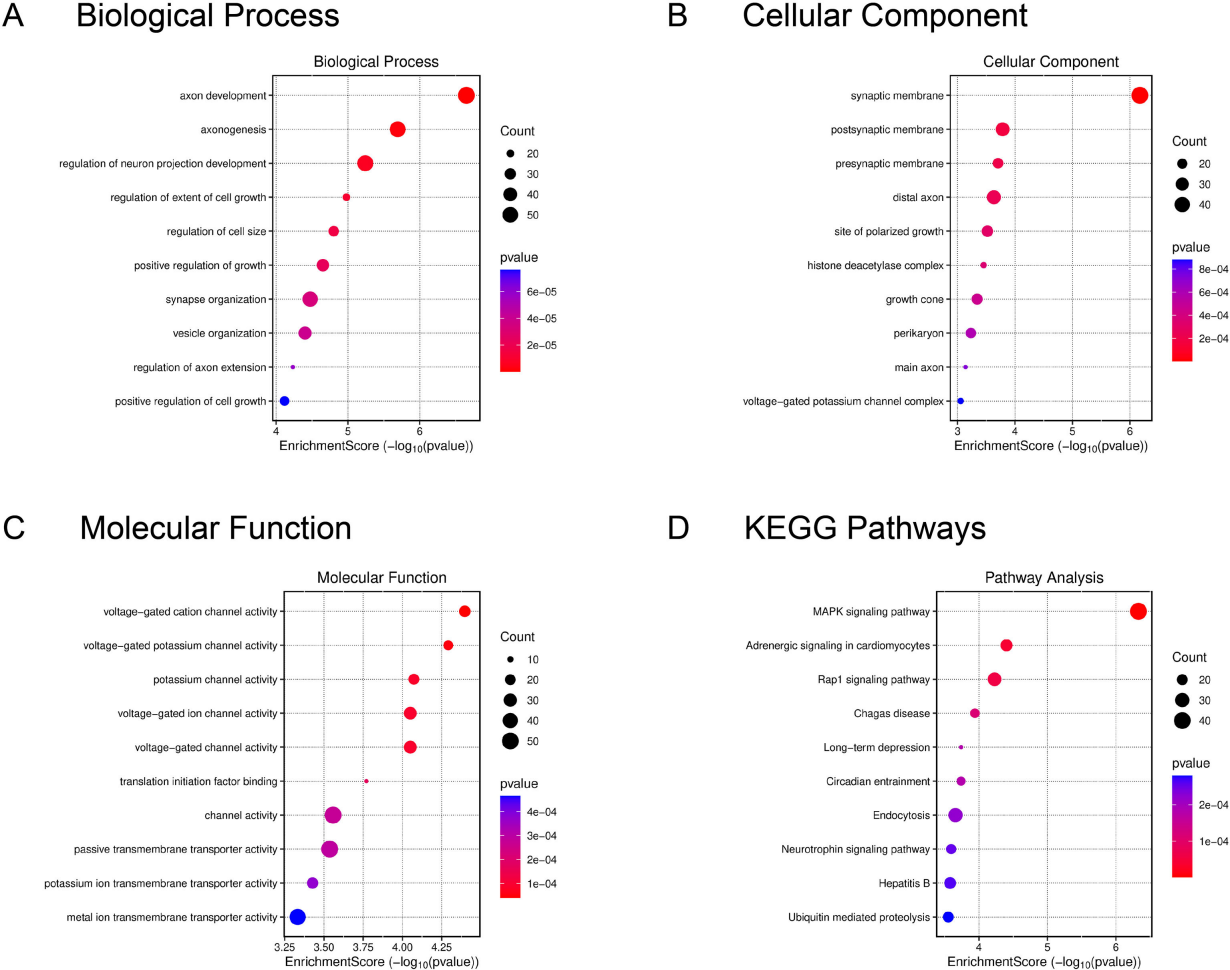
GO functional annotation and KEGG pathway enrichment analysis of target genes for the four miRNAs. **(A)** Biological process, **(B)** Cellular component, **(C)** Molecular function, **(D)** KEGG pathway analysis.

Functional enrichment analysis and annotation of the predicted target genes were conducted using the DAVID database. Genes jointly predicted by two or more miRNAs were screened as candidate target genes. As illustrated in **Figure 6A**, a total of 1, 257 target genes were identified. As illustrated in **Figure 7**, the enrichment results covered biological processes, cellular components, molecular functions, and KEGG signaling pathways. Biological process enrichment analysis revealed that the target genes were significantly enriched in multiple pathways, including axon development, axonogenesis, and regulation of neuron projection development. Cellular component enrichment analysis indicated that the target genes were markedly enriched in multiple cellular structures, including the synaptic membrane, postsynaptic membrane, and presynaptic membrane. Molecular function enrichment analysis demonstrated that the target genes were significantly enriched in multiple functional terms, including voltage−gated cation channel activity, voltage−gated potassium channel activity, and potassium channel activity. KEGG pathway enrichment analysis demonstrated that the target genes were mainly enriched in the MAPK signaling pathway, adrenergic signaling in cardiomyocytes, and the Rap1 signaling pathway.

## Discussion

PC is the second most prevalent malignancy among men worldwide (38, 39) and poses a severe threat to male health globally (40). Statistics show that approximately 1.5 million new cases are diagnosed worldwide each year, leading to 390 000 deaths (1). However, the high false−positive rate of PSA screening remains a major clinical challenge, which causes unnecessary emotional distress, increases medical costs from redundant examinations, and brings potential physical harm to patients (41). Meanwhile, nearly 25% of PC patients present with PSA levels within the normal reference range, thereby causing false−negative outcomes and missed diagnoses (42). Patients may suffer from adverse consequences, including delayed diagnosis, disease progression, poor prognosis, declined quality of life, and elevated treatment costs. Therefore, it is critical to develop more specific biomarkers for PC to address this clinical dilemma. Given their frequent dysregulation in malignant tumors, high tissue specificity, and stable existence in plasma and serum (43), mature miRNAs hold great potential as promising circulating biomarkers for early cancer detection. Numerous studies have demonstrated that mature miRNAs can act as not only diagnostic biomarkers but also potential therapeutic targets for PC (44). This study aimed to identify serum miRNAs with optimal diagnostic efficacy for PC and thus designed a three−phase research protocol. Through sequential screening and validation, a diagnostic panel comprising four specific miRNAs was successfully established. This panel enables more efficient screening and diagnosis of PC (AUC = 0.825; sensitivity = 83.33%, specificity = 69.05%) (**Table 3**).

Porzycki et al. reported that elevated serum miR−141 expression in PC patients could serve as a novel and promising diagnostic biomarker for PC (45). However, their study only focused on a single mature miRNA. In contrast, our study adopted a three−stage approach to screen for mature serum miRNAs with diagnostic potential for PC. Subsequently, statistical algorithms were applied to construct three mature miRNA panels with optimal diagnostic performance. First, following a comprehensive review of relevant literature, we selected 10 PC-associated mature miRNAs as candidate molecules. During the training and validation phases, we observed significantly dysregulated serum levels (miR-101-3p, miR-154-5p, miR-199a-5p, miR-145-5p, miR-30c-5p, and miR-30a-5p) were significantly dysregulated in PC compared to NC. Ultimately, a highly efficient diagnostic panel (AUC = 0.825; sensitivity = 83.33%, specificity = 69.05%) comprising four specific mature miRNAs—miR-101-3p, miR-154-5p, miR-199a-5p, and miR-145-5p—was successfully developed for PC diagnosis.

Among the markers included in this diagnostic panel, miR−145−5p exhibited the highest diagnostic efficacy (AUC = 0.832; 95% CI: 0.629 to 0.772). Armstrong et al. demonstrated that downregulated expression of miR-145-5p may facilitate PC progression by relieving the inhibition of *MYO6* and further inducing EMT (46), indicating its crucial tumor-suppressive role. Luo et al. indicated that miR-145-5p may regulate the TGF-β signaling pathway by suppressing epithelial-mesenchymal transition and tumor growth, thereby inhibiting bone metastasis in PC (47). As reported in our previous studies, miR-145-5p presents distinctly differential expression profiles in normal renal (48) and bladder tissues (49) relative to malignant counterparts, further validating its feasibility as a promising diagnostic biomarker.

Another miRNA investigated by our research group is miR-101-3p. Studies have shown that miR-101-3p can specifically inhibit *TRIB1* expression and abrogate its pro-inflammatory effects, thereby suppressing PC progression (50); thus, it may serve as a novel therapeutic biomarker for PC. Earlier studies have confirmed the potential of circulating miR-101-3p as a diagnostic biomarker for hepatocellular carcinoma (51) and colorectal cancer (52). The present study further clarifies the diagnostic utility of miR-101-3p in PC.

Research has demonstrated that miR−154−5p inhibits proliferation, migration, and invasion by targeting *E2F5* in PC cell lines. Thus, miR-154-5p typically exerts a tumor-suppressive role in PC (53), with significantly downregulated expression in cancerous tissues—consistent with the serum miR-154-5p expression patterns observed in our experiments. In breast cancer, miR-154-5p exerts pro−apoptotic and anti−proliferative effects by targeting *NAMPT* and reducing intracellular NAD levels; additionally, it can enhance cancer cell sensitivity to chemotherapeutic agents, playing a crucial role in regulating tumor progression and chemoresistance (54). Our results revealed that serum miR-154-5p expression was markedly decreased in PC patients, indicating that this miRNA could act as a promising diagnostic biomarker.

In our study, miR-199a-5p was found to be downregulated in the serum of PC patients, which is consistent with the findings reported by Zhong JJ et al. (55). Previous research has revealed that miR-199a-5p exerts a tumor-suppressive role in PC by post−transcriptionally inhibiting *HIF-1α* (56), suggesting that it may serve as a promising non−invasive biomarker for the early diagnosis of PC. In contrast, Gómez-Acebo I et al. reported that miR-199a-5p was significantly overexpressed in the serum of PC patients and acted as an independent marker for elevated PC risk, with a pro-tumorigenic function in PC (57). This evidence highlights the complex and dual role of miR-199a-5p in tumor progression and underscores the necessity of further studies to clarify its molecular mechanisms and clinical potential as a therapeutic target.

*NFASC* was identified as a key common target gene of the four-miRNA panel in our bioinformatics analysis. Du M et al. (58) had found that the 1q32.1 genomic locus, where *NFASC* resides, is a well-established prostate cancer susceptibility region, and noncoding risk variants at this locus regulate *NFASC* expression through long-range chromatin interactions in a prostate tissue*-*specific manner. *NFASC* encodes an L1 family immunoglobulin-like cell adhesion molecule that participates in cell adhesion, membrane organization, and cytoskeleton regulation. In prostate cancer, *NFASC* expression is significantly downregulated in tumor tissues compared with normal controls, suggesting its potential role as a tumor suppressor. Mechanistically, loss of *NFASC* may weaken cell-cell adhesion and facilitate cancer cell migration and invasion, thereby promoting disease progression. Collectively, our findings indicate that the four-miRNA panel may exert its biological effects in prostate cancer by coordinately targeting *NFASC*, highlighting the critical role of NFASC in prostate cancer pathogenesis and supporting its potential as a downstream effector molecule for the diagnostic miRNA signature. We acknowledge that database-derived enrichment results are limited by annotation bias, and NFASC may not capture prostate cancer-specific biology. The regulatory interaction between miRNAs and NFASC, as well as its precise mechanistic contributions, await further experimental validation.

## Conclusion

A panel consisting of miR-101-3p, miR-154-5p, miR-199a-5p, and miR-145-5p exhibits favorable diagnostic performance for PC. This serum miRNA panel possesses considerable translational potential and provides a novel strategy to address two major global clinical challenges: the high false−positive and false−negative rates associated with conventional PSA testing.

## Data Availability

The raw data supporting the conclusions of this article will be made available by the authors, without undue reservation.
