# Supplementary material for: A four-miRNA serum panel as a diagnostic biomarker for prostate cancer: an exploratory and validation study
Source: Front Oncol. 2026 Jul 27;16:1866712. doi: 10.3389/fonc.2026.1866712 (PMC13453685; doi:10.3389/fonc.2026.1866712)
Supplement: Supplementary file 1 [file Table1.docx]

| Supplementary Table 1. Outcomes of receiver operating characteristic curves for different panels. | | | | | | |
| --- | --- | --- | --- | --- | --- | --- |
|  | AUC (95% CI) | P value | Youden index | Cut-off | Sensitivity | Specificity |
| 5-miRNA panel | 0.850 (0.787 to 0.901) | < 0.0001 | 0.5833 | >0.5129 | 79.76% | 78.57% |
| Best of 4-miRNA panels |  |  |  |  |  |  |
| Panel 1 | 0.825 (0.759 to 0.879) | < 0.0001 | 0.5238 | >0.4332 | 83.33% | 69.05% |
| Panel 2 | 0.836 (0.771 to 0.889) | < 0.0001 | 0.6071 | >0.5226 | 82.14% | 78.57% |
| Panel 3 | 0.791 (0.721 to 0.849) | < 0.0001 | 0.5000 | > 0.5411 | 72.62% | 77.38% |
| Panel 4 | 0.819 (0.752 to 0.874) | < 0.0001 | 0.5476 | >0.5627 | 72.62% | 82.14% |
| Panel 5 | 0.832 (0.767 to 0.885) | < 0.0001 | 0.5476 | >0.6006 | 69.05% | 85.71% |
| Best of 3-miRNA panels |  |  |  |  |  |  |
| Panel 1 | 0.771 (0.700 to 0.832) | < 0.0001 | 0.4524 | >0.5266 | 70.24% | 70.24% |
| Panel 2 | 0.805 (0.737 to 0.862) | < 0.0001 | 0.5119 | >0.5432 | 72.62% | 78.57% |
| Panel 3 | 0.770 (0.699 to 0.831) | < 0.0001 | 0.4286 | >0.6152 | 55.95% | 86.90% |
| Panel 4 | 0.807 (0.739 to 0.864) | < 0.0001 | 0.5119 | >0.5280 | 73.81% | 77.38% |
| Panel 5 | 0.757 (0.685 to 0.820) | < 0.0001 | 0.3929 | >0.5215 | 69.05% | 70.24% |
| Panel 6 | 0.803 (0.735 to 0.860) | < 0.0001 | 0.4643 | >0.5493 | 67.86% | 78.57% |
| Panel 7 | 0.793 (0.724 to 0.852) | < 0.0001 | 0.4643 | >0.5056 | 73.81% | 72.62% |
| Panel 8 | 0.775 (0.704 to 0.836) | < 0.0001 | 0.4643 | >0.5416 | 70.24% | 76.19% |
| Panel 9 | 0.774 (0.703 to 0.835) | < 0.0001 | 0.4524 | >0.5317 | 67.86% | 77.38% |
| Panel 10 | 0.799 (0.731 to 0.857) | < 0.0001 | 0.4762 | >0.4926 | 76.19% | 71.43% |
| AUC: area under curve; CI: confidence interval. | | | | | | |
